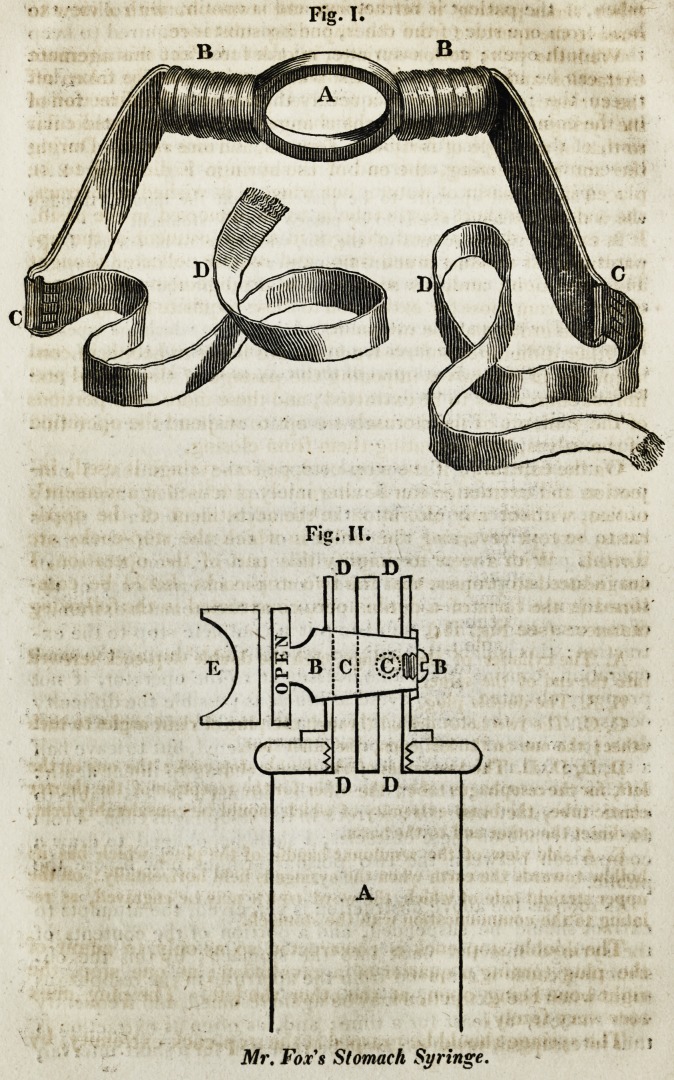# Observations on the Use, Together with a Proposed Alteration in the Construction, of the Syringe for Extracting Poisons from the Human Stomach

**Published:** 1825-03

**Authors:** Francis Fox

**Affiliations:** Derby.


ART. X.—*Observations on the Use, together with a proposed Alteration in the Construction, of the Syringe for extracting Poisons from the Human Stomach.* By FRANCIS FOX, jun. M.D. Derby.

WE are encouraged to believe, from recent experience, that the application of the syringe for the purpose of washing out the stomach in cases of poisoning, will frequently lead to the preservation of human life; where, without the adoption of such a measure, the medical attendant would remain an almost passive spectator during the consequent fatal train of symptoms. It is from a strong impression, founded on a practical knowledge of the utility of the instrument, that the following observations are offered to the public, with a view to elucidate the important points in its construction, and to render its successful operation more certain.

The patient is most manageable in the recumbent posture. The first obstacle which occasionally presents itself, arises from the teeth being firmly closed together: after this has been obviated, by introducing a wedge of wood or iron, there is great management and attention required to prevent the mouth from being closed again; and, in those instances where there are but few assistants in the operation, the permanent accomplishment of this point is almost impracticable by the usual mode of holding a piece of wood between the teeth at one side of the mouth, when, if the patient is refractory, and is continually rolling the head from one side to the other, one assistant is required to keep the mouth open; and, even after all the force and management that can be had recourse to, the wooden gag will slip from between the teeth, and consequently the operator will be foiled by the compression, and perhaps more thoroughly by the division, of the œsophagus tube. This very serious obstacle might be removed by using an iron bit, as shown in Fig. I. page 210.

A. The central aperture to be placed within the mouth, through which the œsophagus tube is to be passed into the stomach.

B, B. The two side parts of the bit, which are to be curved backwards towards the ears, to insure the proper position of the aperture A. They are to be covered with tape or leather, for the teeth to bear against.

C, C. The rings at the extremities of the bit, to which the tapes D, D, are fastened. These tapes are to be fastened behind the head, and tied at the upper and back part of the neck, so as to keep the bit permanently fixed in the mouth.

The effects of this instrument are too evident to require further explanation.

Without detailing the several steps of the operation, I now pass on to state that, after having injected a sufficient quantity of water, or other liquid, into the stomach, the order of things has to be reversed, and the contents of the stomach extracted. To this part of the process impediments arise, from fibrous or coagulated substances, and also from the internal coat of the stomach itself obstructing the aperture at the stomach extremity of the œsophagus tube, so as to put a complete stop to the extraction: this is liable to happen several times during the same operation, causing great embarrassment to the operator, if not properly obviated. To avoid as much as possible the difficulty occasioned by the stomach, it is not advisable to attempt to withdraw the whole of the liquid previously injected, but to leave half a syringe full in the stomach; then proceeding to inject again: by this precaution, the inside of the stomach is less liable to come in contact with the aperture in the œsophagus tube. From the occurrence of these obstacles, I shall be obliged to draw a comparison between the two poison-syringes now before the public.

As soon as the above obstruction is perceived, the attempts to extract should be suspended, and a portion of the contents of the syringe injected back into the stomach: by this the obstructing cause is removed from the aperture in the œsophagus tube, when the extraction may be again resumed, and frequently with success, at least for a time; and, as often as extraction is thus interrupted, injection must be repeated for a short interval,

Fig. I.

Fig. II.

*Mr. Fox's Stomach Syringe.*

occasionally withdrawing the œsophagus tube a little, and at other times passing it further into the stomach, with a view to remove the aperture from the impeding substance.

With the valve poison-syringe, in order to effect this alternate extraction and injection, the œsophagus tube must be taken off the syringe, and refixed in a reversed position: the direction of the syringe is also advised to be changed from the perpendicular to that of an angle of forty-five degrees, and *vice versâ*. During the act of injecting, the end of the syringe is directed to be placed in the basin of water; but when it is wished to extract, the end of the short elastic tube is to be immersed in the basin. It is evident these several changes in the adjustment of the apparatus must exhaust much time, and require collected thought and attention; and, as soon as the arrangements have been changed from those for extraction to those requisite for injection, a reverse order may be necessary.

Other difficulties and obstructions are liable to occur, in consequence of the valves impeding the passage of the clotted and fibrous substances to be extracted; and these more solid portions of the contents of the stomach are apt to suspend the operation of the valves, by preventing them from closing.

On the contrary, if the stop-cock poison-syringe is used, injection and extraction can be alternately practised at a moment's notice, without any alteration in the adjustment of the apparatus, except reversing the order in which the stop-cocks are turned. With a view to simplify this part of the operation, I am induced to propose that the two stop-cocks should be combined in the form of a double one, constructed in the following manner: (see Fig. II.)

A. The cylinder of the syringe, with the double stop-cock screwed into the end of the same.

B, B. The double plug.

C, C. The two holes drilled through the plug at right angles to each other; the one on the left open, the other shut.

D, D, D, D. The two boxes of the double stop-cock: the one on the left, for the œsophagus tube; the other for the reception of the shorter elastic tube, the brass extremity of which should be considerably bent, to direct the other end to the basin.

E. A side view of the semilunar handle of the plug, which has its hollow towards the earth when the syringe is held horizontally; on the upper straight side of which, the word OPEN may be engraved, as relating to the communication with the stomach.

The double stop-cock is constructed so as only to admit of the plug turning a quarter of a revolution; at one stop, the right bone being open, at the other the left. The plug must turn very freely.

The syringe should be grasped at the stop-cock extremity, by the left hand; and, by the flexion and partial extension of the fore-finger, placed in the semilunar handle of the plug, the communication is alternately formed between the syringe and the œsophagus tube, and between the syringe and the short tube immersed in the basin; the right hand is kept on the handle of the syringe.

In order to manage the double stop-cock, the attention has only to be directed to these two rules:—

*To inject*, the word OPEN must be upwards, only whilst the piston-rod is forced into the syringe.

*To extract*, the word OPEN must be upwards, only whilst the piston-rod is drawn out.

To explain the operation of this stop-cock more fully:—during injection, the straight side of the semilunar handle must be uppermost whilst the piston-rod is forced into the syringe, after which the stop-cock must be moved to the other stop, by the partial extension of the finger, and the piston-rod drawn out again, and so on by turns. In extracting, the straight side of the semilunar handle must be uppermost whilst the piston-rod is drawn out, the stop-cock being then moved by the partial extension of the finger, and the piston-rod forced back again.

In concluding what I have to say on the construction of the poison-syringe, I have only to add, that the œsophagus tube should be as large as the nature of the œsophagus will admit of; the bore of the stop-cocks should be quite equal to the calibre of the œsophagus tube; and it is a question whether the stomach end of this tube should not be quite open, and not formed like a catheter, as is now usually done, as it is highly desirable that the clotted and fibrous contents of the stomach should pass through the instrument as freely as possible. The syringe should hold twelve ounces at least.

Convinced, as I am, of the superiority of the stop-cock syringe over the one constructed with valves, for extracting poison from the stomach, I feel anxious that those who are about to apply the syringe, should not fail in their attempts, by using an instrument which will not easily and quickly surmount all the obstacles liable to occur during an operation of so much importance to the individual on whom it is performed: but it should be thoroughly understood that the valve syringe is only objectionable in the operation of evacuating the contents of the stomach; in all other instances, for the purposes of injecting, it is an extremely applicable and elegant apparatus.

In conclusion, allow me to assure you that it will be a source of future gratification to me, should these remarks lead to a more successful application of that principle which they are intended to promulgate and defend.

## Figures and Tables

**Fig. I. Fig. II. f1:**